# Two separate neural pathways, lateral and medial, for sensory decisions in mammals: switching of attention between the outer and inner cognitive worlds

**DOI:** 10.3389/fnins.2025.1640801

**Published:** 2025-07-04

**Authors:** Kensaku Mori, Hitoshi Sakano

**Affiliations:** ^1^RIKEN Center for Brain Science, Wako, Saitama, Japan; ^2^Department of Brain Function, School of Medical Sciences, University of Fukui, Matsuoka, Fukui, Japan; ^3^Department of Animal Behaviors, School of Veterinary Medicine, University of Tokyo, Bunkyo-ku, Tokyo, Japan

**Keywords:** cortical domain, attentional switching, outer-world cognition, inner-world cognition, somatic and emotional motor outputs

## Abstract

Mammalian sensory cortices detect changes both inside and outside of the body. They identify sensory information from the surrounding world, evaluate the current situation, and generate top-down signals to induce emotional and behavioral outputs. The cortices also detect physiological changes inside of the body, such as internal pain, thirst, fever, and retronasal odors. Thus, the cortical attention is directed to either the outside or inside of the body. As consciousness seems to be generated by sensory stimuli together with the recollected memory scene, self-cognition may be divided into two categories: one for the outside world and the other for the inside world. We have previously proposed that in the mammalian olfactory system, orthonasal and retronasal odor signals are separately detected in the inhalation and exhalation phases during respiration by the lateral and medial parts of the olfactory bulb, respectively. We further speculated that orthonasal and retronasal olfactory information are transmitted to the higher-order cognitive areas by the lateral pathway for outer-world information and medial pathway for inner-world information, respectively. In the present article, we propose that the late exhalation phase provides the time frame for generating internal attention and internal signals for behavioral and emotional outputs. We will discuss how the recognition of external objects is combined with internal emotion to generate associative memory of object-feeling, namely emotional episodic memory. It will also be discussed how the two types of attention directed toward the outer and inner worlds are switched from one to the other to reset self-cognition and consciousness.

## Introduction

When animals are awake, they pay attention to the surrounding world to seek food, to avoid dangers, and to find mating partners for the survival of individuals and species. Sensory stimuli activate associated memory with its valence to evaluate the current situation and to make appropriate output decisions. Sensory systems detect subtle changes not only in the outside environment, but also in the inner body. When we are relaxed and do not have to focus on the outer world, attention is directed to the inner world to find whether the current situation is satisfactory or not (Chun et al., 2011). It is assumed that consciousness is formed based on the sensory inputs from both outside and inside of the body (Craig, 2002; Azzalini et al., 2019). Our consciousness is also generated by spontaneous firing of memory engrams in the self-cognitive world longing for the past (Gallagher, 2000). Thus, our attention is frequently switched from the outer world to the inner world, or vice versa, moving our consciousness back and forth between the two cognitive worlds.

**Figure 1:**
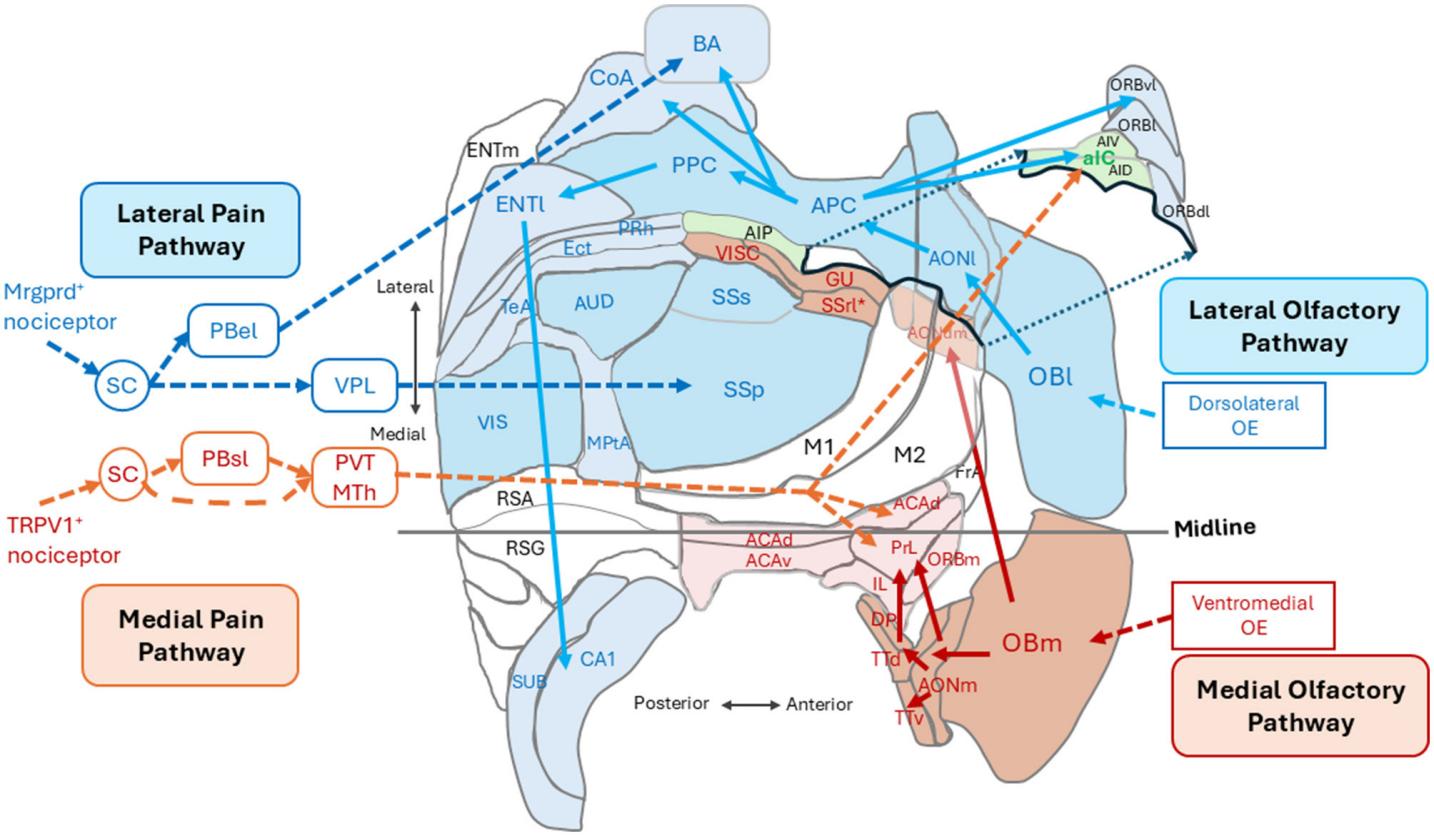
An unfolded map of the left cerebral cortex in the mouse (dorsal view) showing the spatial arrangement of cortical areas for the outer-world cognition (in blue) and those for the inner-world cognition (in magenta and orange). The lateral olfactory pathway (blue arrows) processes orthonasal/exteroceptive odor information and transmits it form the lateral map of the olfactory bulb (OBl) to the anterior and posterior piriform cortices (APC and PPC) via the lateral part of the anterior olfactory nucleus (AONl) and further to the higher outer-world cognitive areas. The medial olfactory pathway (magenta arrows) processes retronasal interoceptive odor information and transmits it from the medial map of the olfactory bulb (OBm) via the medial part of the anterior olfactory nucleus (AONm) and tenia tecta (TTd and TTv) to the higher inner-world cognitive areas, i.e., the medial prefrontal cortex (shown in pink). The lateral pain pathway (blue broken-line arrows) originates from the Mrgprd-positive nociceptors and transmits exteroceptive pain information to the somatosensory cortex (SSp) via the ventral posterolateral nucleus (VPL) of the thalamus and to the basal amygdala (BA) via the external lateral parabrachial nucleus (PBel). The medial pain pathway (orange broken-line arrows) originates from the TPPV1-positive nociceptors and transmits interoceptive pain information to the anterior insular cortex (aIC) and to the higher inner-world cognitive areas including the anterior cingulate area (ACA) and prelimbic cortex (PrL) via the thalamic paraventricular nucleus (PVT) and medial thalamic nuclei (MTh). See glossary for abbreviations. Adopted from Mori and Sakano (2022a).

How is it, then, that these two sets of sensory information, one from the outer world and the other from the inner world, are processed in the sensory cortices for decision making? In the mammalian olfactory system, orthonasal and retronasal odorants are separately detected in the inhalation and exhalation phases of the respiratory cycle, respectively (Mori and Sakano, 2011, 2022a). Each olfactory bulb (OB), right and left, has two mirror-symmetrical glomerular maps, lateral and medial that may possess different functions. It has been a long-standing question why the olfactory maps are duplicated in each OB. Do they possess different functions, or are they just spares? One possibility is that the lateral map detects orthonasal odor signals and the medial map detects retronasal odor signals. We speculated that the orthonasal and retronasal odor information is separately transmitted to the higher-order cognitive regions through the lateral pathway for outer-world and the medial pathway for inner-world, respectively (Figure 1; Mori and Sakano, 2022a,b). In this perspective article, we will discuss how these two sets of sensory information, lateral and medial, are processed and combined in the cerebral cortices for generating the object images and internal emotion.

We also proposed that olfactory detection, perception, and decision making are closely related to the respiratory cycle (Narikiyo et al., 2018; Mori and Sakano, 2021, 2022a, 2024a,b, 2025). It appears that informational processing in other sensory systems also coordinates with the olfactory system to integrate all sensory inputs from the target object (Mori and Sakano, 2025). Although detection and transmission of orthonasal and retronasal odor information are correlated with the inhalation and exhalation phases, respectively, it is not clear yet whether the processing of olfactory information in the higher cognitive areas is correlated with the respiratory cycle. It will be interesting to study how the consciousness is switched between the outer world and inner world during the respiratory cycle.

When we are awake, we look at ourselves as an object and evaluate the situation in the surrounding world. Our attention is directed toward the self, not only in the outer world but also in the inner cognitive world. We ask ourselves whether the current situation is satisfactory to us. If not, we are motivated to improve the situation by trying to achieve a more ideal state. In humans, the internal language plays an important role in evaluating the current status and constructing the strategy for behavioral outputs. Language is a useful tool not only in communicating with other individuals for smooth social interactions but also in thinking about the object including ourselves in the inner world. We as first-person, talk to the third-person self in the cognitive world for thinking. In this perspective article, we would like to discuss the language in the context of consciousness and self-cognition in humans.

## Outer-world lateral pathway and inner-world medial pathway

The mammalian brain classifies pain information into two different categories: one is external pain that induces reflexive-defensive behaviors such as quick withdrawal of the paw when it touches a hot plate, and the other is internal pain that induces affective motivational behaviors such as licking an injured paw to soothe the suffering (Price, 1999; Kulkarni et al., 2005; Bushnell et al., 2013; Huang et al., 2019; De Ridder et al., 2022; Wang et al., 2022). It has been demonstrated that external pain and internal pain are detected by different nociceptors and conveyed to the cortex by separate neural pathways (Huang et al., 2019; De Ridder et al., 2022; Wang et al., 2022; Wercberger and Basbaum, 2019). The lateral pain pathway (Figure 1, blue broken-line arrows), originating from the Mrgprd-positive nociceptors, processes external pain information that induces reflexive-defensive behaviors. The lateral pathway transmits external pain to the somatosensory cortices (SSp and SSs) via the ventral posterolateral nucleus (VPL) of the thalamus and to the basal amygdala (BA) via the external lateral parabrachial nucleus (PBel; Figure 1; Huang et al., 2019; De Ridder et al., 2022).

The medial pain pathway (Figure 1, orange broken-line arrows), originating from the TRPV1-positive nociceptors, processes internal pain information that induces affective motivational behaviors. The medial pathway transmits internal pain information to the anterior insular cortex (aIC) and higher inner-world cognitive areas, including the anterior cingulate area (ACA) and prelimbic cortex (PrL), via the thalamic paraventricular nucleus (PVT) and medial thalamic nuclei (MTh). In the human cortex, the aIC is a hub area of the salience network encoding the behavioral relevance (saliency) of sensory stimuli (Seeley et al., 2007). In contrast, the ACA and PrL in the medial prefrontal cortex are key stations in the default mode network (DMN; Raichle et al., 2001; Greicius et al., 2003; Buckner et al., 2008) that is responsible for generating negative emotional and autonomic responses to the pain (De Ridder et al., 2022). Thus, external pain and internal pain are separately processed by different cortical sensory areas and different higher cognitive areas.

**Figure 2:**
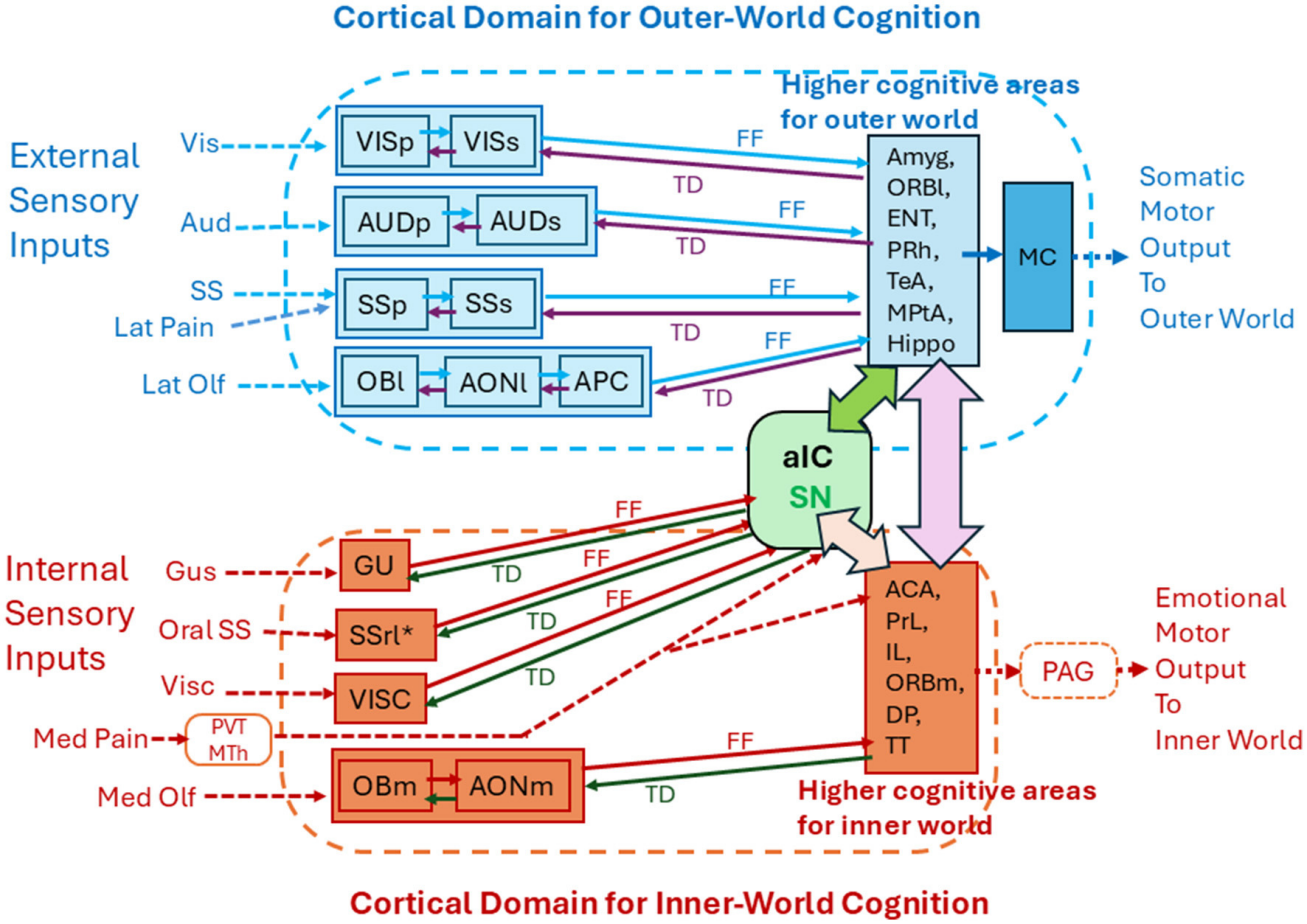
Functional subdivision of the cortical areas into the outer-world cognitive domain and inner-world cognitive domain. The cortical domain for the outer-world consists of the exteroceptive sensory cortices (visual, auditory, somatosensory, and orthonasal olfactory cortices) and the higher-order multisensory cognitive areas (Amyg, ORBl, ENT, PRh, TeA, MPtA, and Hippo). The outer-world cognitive domain connects with motor cortices (MC) to generate somatic motor output. The cortical domain for the inner-world consists of the interoceptive cortical areas (gustatory, visceral, intraoral somatosensory, and retronasal olfactory cortices) and the higher-order emotion-generating cognitive areas (ACC, PrL, IL, ORBm, DP, and TT). The inner-world cortical domain connects with the periaqueductal gray (PAG) in the midbrain to generate emotional motor output. The anterior insular area (aIC) detects salience signals and may instruct switching of the two types of attention that are directed toward the outer and inner worlds. Solid arrows indicate corticocortical connections. FF indicates feedforward transmission of sensory signals. TD indicates top-down transmission of cognitive scene signals. See glossary for abbreviations.

Figure 1 illustrates the spatial arrangement of cortical sensory areas that process outer-world information (OBl, AONl, APC, PPC, SSp, SSs, AUD, and VIS, shown in dark blue) and higher multisensory cognitive areas possibly responsible for outer-world cognition (ORBl, ORBvl, CoA, BA, ENTl, PRh, ECT, TeA, MPtA, CA1, and SUB, shown in pale blue). In the human cortex, these areas correspond to the exteroceptive sensory cortices and frontoparietal central executive network (FPN; Vincent et al., 2008). Figure 1 also illustrates the spatial arrangement of cortical sensory areas that process inner-world information (OBm, AONm, GU, VISC, SSrl^*^, shown in magenta) and higher multisensory cognitive areas responsible for inner-world cognition (aIC, ACAd, PrL, IL, ORBm, and DP/TTd). It should be noted that in both rodents and humans, the higher key areas for outer-world cognition (FPN in humans) are assembled in the lateral, temporal, parietal, and occipital regions of the cortex, whereas the higher key areas for inner-world cognition gather together in the medial midline regions (DMN in humans) or in the anterior insular cortex (SN in humans). These considerations suggest that there may be three distinct cognitive domains in the cortex: the first one is the cortical domain for outer-world cognition (exteroceptive sensory areas and higher cognitive areas for the outer world), the second one is the cortical domain for inner-world cognition (interoceptive sensory areas and higher cognitive areas for the inner world), and the third one is the anterior insular domain which is a key player in switching the attentional target from the outer world to the inner world, or vice versa, as will be discussed in the next section (Figure 2).

## Switching of attention and consciousness

Appropriate switching of attention between the outer and inner worlds is needed to make adaptive behavioral responses to constantly changing environmental and internal situations (Chun et al., 2011; Katsuki and Constantinidis, 2014; Nani et al., 2019). For example, when animals search for food, their attention is directed toward the outer world. Once they find food and put it in their mouth, attention is switched to the inner world considering whether it should be swallowed or not. What would be the neural circuit mechanisms for paying attention to the outer and inner worlds and switching attention between them?

We previously hypothesized that cognition of olfactory imagery of an object depends not only upon the odor-induced firings of pyramidal cells in the olfactory cortex but also subsequent replay-firing of the same set of pyramidal cells by the top-down cognitive-scene signals generated in higher cognitive areas (Mori and Sakano, 2025). We further hypothesized that during late exhalation, the top-down signals are simultaneously transmitted back to all the olfactory, visual, somatosensory, and auditory cortices to induce replay-firing of pyramidal cells in each sensory cortex (Mori and Sakano, 2025). Thus, we speculate that multisensory cognition of an external object is mediated by not only the feedforward activation of pyramidal cells but also generation and top-down transmission of cognitive scene-signals from the higher cognitive areas to each sensory cortex. In other words, the cognition process of external objects requires two temporal stages. The first stage includes feedforward processing of sensory information in each sensory cortex and transmission of processed signals to higher multisensory cognitive areas. In the olfactory cortex for the lateral pathway, the first stage occurs only during inhalation, although it could occur at any time in the visual, auditory, and somatosensory cortices. The second stage is the generation of cognitive scene-signals by the higher cognitive areas and simultaneous top-down transmission to each sensory cortex. We hypothesize that the second stage may occur only during the late exhalation phase.

As stated above, recognition of olfactory objects requires feedforward transmission of olfactory signals and top-down transmission of cognitive scene signals in the circuits of the olfactory cortex and higher cognitive areas for the outer world (Figure 2). By the same token, recognition of visual objects may require feedforward and top-down transmission in the circuits of the visual cortex and higher cognitive areas. Recognition of auditory objects may require feedforward and top-down transmission in the circuits of auditory cortex and higher cognitive areas. Thus, when animals engage in tasks that require object recognition, feedforward sensory transmission and top-down cognitive-scene transmission regularly occur in the cortical domain for outer-world cognition (Figure 2).

In humans, the anterior insular cortex (aIC) is a key structure of the salience network (SN) and acts as a switch for the default mode network (DMN) and frontoparietal central executive network (FPN; Sridharan et al., 2008; Menon and Uddin, 2010; Goulden et al., 2014). FPN is active when a person engages in tasks that require object recognition and interaction with the outer world. In contrast, DMN is active when the person does not interact with the outer world and attends to the inner cognitive world (Fox et al., 2005; Menon, 2023). The human SN is assumed to have a switching function between the outer-world attention and inner-world attention (Menon and Uddin, 2010). In rodents, the aIC consists of ventral agranular insular cortex (AIV) and dorsal agranular insular cortex (AID; Figure 1). Optogenetic stimulation of aIC neurons induces suppression of DMN activity (Menon et al., 2023), suggesting a functional role of the rodent aIC in switching attention.

## Roles of attention to the outer world and inner world

During wakefulness, the mammalian brain receives a barrage of multisensory information from both the outer and inner worlds (Zaidel and Salmon, 2023). One of the key roles of attention to the outer world is to filter the barrage of information and selectively enhance the cortical cognition of external objects so that behavioral decisions can be made quickly (Chun et al., 2011). Neural circuit mechanisms to generate attention to the external world are not well-understood. However, possible candidates may be diffuse axonal projections of neurons in the basal forebrain or claustrum/endopiriform nucleus to the cortical domain for outer-world cognition (Figure 2; Behan and Haberly, 1999; Zingg et al., 2012; Xu et al., 2015; Do et al., 2016; Villano et al., 2017; Narikiyo et al., 2020; Wang et al., 2021). Diffuse projections suggest that these neurons are involved in setting general levels of activation of all neurons in the target domain. We speculate that these diffuse axonal projections may control the levels of attention in these cortical areas to facilitate processing of feedforward sensory information and cognizing objects in the outer world.

Attention is directed not only to the entire outside world but also to a particular object within it. When animals hear the voice of a predator from a distance, they pay attention to the direction of the voice and generate visual imagery of the predator based on learned memory. Voice-induced generation of predator imagery in the higher cognitive areas may play a key role in paying top-down attention to the predator. Top-down transmission of the imagery to the visual cortices may selectively depolarize the dendrites of pyramidal cells that are tuned to the visual inputs of the predator and, thus facilitate detection of visual signals from the predator. As described above, cognition of an external object is generated based on both feedforward sensory processing and top-down cognitive-scene transmission. We hypothesize that top-down transmission of cognitive scene-signals directs the top-down attention to the object.

We propose that when the animal pays attention to a particular object, the cerebral cortex internally generates top-down cognitive scene signals and transmits them back to the sensory cortices. Even when the object is not cognized in the outer world, the cortex can attend to the object by spontaneous recollection of multisensory memory of object imagery in the higher cognitive areas. The self-generated scene signals are transmitted back to the sensory cortices to facilitate object cognition. We speculate that scene information is internally generated during the late exhalation phase. In other words, top-down internal attention to a specific object appears to be generated during the late exhalation phase.

One of the key functions of attention to the inner world may be to facilitate cortical processing of body-state information so that the internal body-state cognition is promoted to induce emotional responses. We speculate that attention to the inner world is controlled by diffuse axonal projections of neurons in the basal forebrain and claustrum/endopiriform nucleus to the cortical domain for inner-world cognition (Zingg et al., 2012; Do et al., 2016; Narikiyo et al., 2020; Wang et al., 2021; Aguilar and McNally, 2022). We also speculate that top-down internal scene-signals may be generated in the higher cognitive areas, e.g., medial prefrontal cortex, for the inner world during the late exhalation phase, and that they are transmitted back to the interoceptive sensory areas via the top-down pathways (Figure 2). It is possible that top-down transmission of emotional scene signals mediates the content of attention, i.e., attention to a particular emotional state of the self. Attention to a specific emotional state can be triggered by interoceptive sensory signals such as internal pain to induce emotional scene-signals in the higher inner-world cognitive areas. In addition, attention to the emotional state can be intrinsically generated by spontaneous recollection of emotional scene-signals in higher inner-world cognitive areas and top-down transmission of signals to the interoceptive sensory cortices. Attention to a particular emotional state may occur during the late exhalation phase.

External attention and internal attention are distinguished by the types of information over which attention operates in awareness (Chun et al., 2011; Zaidel and Salmon, 2023). External attention focuses on the sensory information originating from the external world. In the olfactory system, external attention appears to be linked to the active inhalation phase (Mori and Sakano, 2024a). Internal attention focuses on the interoceptive sensory information generated in the self-body as well as on the cognitive information internally generated within the self-brain. In other words, the target of internal attention includes top-down cognitive scene-information and emotional scene-information. Internal attention appears to be associated with the exhalation phase because processing of interoceptive/retronasal odor information occurs during the exhalation phase, and top-down processing of cognitive/emotional scene-information occurs during the late exhalation phase (Mori and Sakano, 2024a,b, 2025).

## Associative learning of external objects with internal emotion

We previously proposed that the late exhalation phase provides the temporal dimension for generating top-down cognitive scene-signals in the outer-world cortical domain. We also proposed that associative memory of external sensory inputs with the multisensory object-scene is generated by interactions between the burst firings of pyramidal cells induced by sensory inputs and subsequent replay-firings of the same subset of pyramidal cells induced by top-down cognitive scene-signals (Mori and Sakano, 2025). Here, we further propose that the late exhalation phase provides the time frame for generating top-down emotional scene-signals in the inner-world cortical domain. We also propose that associative memory of internal sensory inputs with the emotional scene is generated by interactions between the burst firings of pyramidal cells induced by inner sensory inputs and subsequent replay-firing of the same subset of pyramidal cells by top-down emotional scene-signals. In accord with this idea, the higher cognitive areas for the inner world, i.e., areas in the medial prefrontal cortex, play a key role in controlling emotional and autonomic outputs (Price, 1999; McKlveen et al., 2015; Skog et al., 2024).

Then, what is the neural circuit mechanism for associative learning of external objects with internal emotion? Let's assume that an animal finds food in the environment. During this period, the outer-world cortical domain may form cognitive imagery of external food during the late exhalation phase. Let's also assume that the animal then eats food. During this period when food is in the mouth, the inner-world cortical domain may form emotional imagery of food, such as tastiness during the late exhalation phase. Because replay firings of pyramidal cells in the outer-world cortical domain may last even after the animal puts food into the mouth, replay firings of pyramidal cells in the inner-world cortical domain may simultaneously occur with replay-firings of pyramidal cells in the outer-world cortical domain during the late exhalation phase. In other words, the generation of emotional imagery of food in the inner-world cortical domain may simultaneously occur with the generation of cognitive imagery of external food in the outer-world cortical domain (Figure 2). Simultaneous burst firings of pyramidal cells in the inner-world domain and in the outer-world domain may result in plastic changes in the synaptic connections between these two populations of pyramidal cells, so that the animal can establish associative learning of external object-cognition with internal emotion-cognition. After associative learning is fully established, the animal can recall the emotional scene of food just by looking at food in the outer world.

As shown in Figure 2, higher cognitive areas in the outer-world domain interact with higher cognitive areas in the inner-world domain directly by cortico-cortical axonal connections or indirectly via cortico-thalamo-cortical connections (Figure 2, pink double-headed arrow). The amygdala and hippocampus in the outer-world domain have direct and reciprocal connections with the areas in the medial prefrontal cortex in the inner-world domain (Marek et al., 2013; Griffin, 2015; Chao et al., 2020; Gangopadhyay et al., 2021).

## Immobility in resetting consciousness

When we are awake, consciousness is constantly “ON.” However, when the self is recognized in a new situation, attention is re-directed to a new object. Animals demonstrate freezing when they encounter a potential danger such as predators. Upon encountering a predator or enemy, rodents show two types of behavioral responses: one is active coping behaviors, fight or flight, and the other is passive coping behaviors such as freezing without showing any movements to avoid the crisis (Behbeharni, 1995; Tovote et al., 2016). Rodents attend to the outer world during the active coping behavior, whereas they switch their attention to the inner world during the passive coping behavior. Freezing is a typical fear response to reset consciousness and allows the animal to avoid danger. It has been thought that freezing is a strong stress response in a dangerous situation. Interestingly, however, freezing is not accompanied by an increase in stress hormones, e.g., adreno-corticotropic hormone (ACTH; Saito et al., 2017). Freezing is “immobility” that is an active decision to reset the behavioral strategy. This allows the rodents to have time for reconstructing a behavioral strategy to escape from the crisis.

Fox odor, trimethyl-thiazoline (TMT), induces fear responses in rodents. In the mouse, TMT activates more than 20 different glomeruli in the dorsal region of the OB that contains two subdomains D_I_ and D_II_: D_I_ is for avoidance and D_II_ is for freezing (Kobayakawa et al., 2007). We have identified a glomerulus for the TMT-responsive olfactory receptor Olfr1019 in the D_II_ subdomain (Saito et al., 2017). Photo-activation of the knock-in (KI) mouse of channel rhodopsin for the *Olfr1019* gene induced freezing, but not avoidance. Plasma concentrations of ACTH did not increase in the photo-illuminated KI mice. Furthermore, the knock-out mouse of Olfr1019 failed to induce freezing but demonstrated avoidance behaviors toward TMT. These observations indicate that freezing (immobility) in danger is not a stress response, but a decision not to move. Moving mice frequently stop their movements, re-setting their consciousness and re-directing their attention, to adapt to the new situation. We assume that immobility is an active response allowing animals to reset their behavioral strategy.

## Language communication in the outside world and inside world

Mammals demonstrate “vocal call” and “call back” behaviors that are primitive social communication among the group members (Seyfarth and Cheney, 2010; Takahashi et al., 2013; Poliva, 2015). When the vocal call is heard, an individual animal attends to the outer world to facilitate making detections and processing auditory information. In contrast, when the call back is generated, the animal attends to the inner world to prepare the return message and executes the vocal emission. Humans also show similar attentional switching in their dialogue and language communication. During the listening phase, the human brain attends to the outer world and concentrates on receiving the vocal word message. After hearing it, the human brain is switched to a word-producing mode, attending to the inner world. In synchrony with this switching, the human brain changes the respiration pattern from quiet respiration to the intentional vocalization-supporting mode of exhalation increasing the laryngeal pressure to produce vocalization (Demartsev et al., 2022).

The anterior cingulate cortex, insular cortex, and orbitofrontal cortex play a key role in instructing the circuitry of midbrain periaqueductal gray (PAG) to generate vocalization and the vocalization-supporting exhalation (Jürgens, 2002; Holstege, 2014; Holstege and Subramanian, 2016; Zhang and Ghazanfar, 2020; Mori and Sakano, 2024b). The cortical pathways to the PAG are also critical in generating emotional behaviors and in controlling the respiratory pattern as well as autonomic outputs. Thus, they are known as the emotional motor system (Holstege and Subramanian, 2016).

It should be noted that attention to the inner world can be divided into the initial “thinking period” and later “vocal word producing period.” During the thinking period, the human brain may internally recall relevant memory and prepare to generate its own message. During the vocal word producing period, the human cortex may exert top-down instruction to the brainstem circuitry for voice production (Jürgens, 2002; Poliva, 2015; Zhang and Ghazanfar, 2020). We speculate that the human brain has evolved to use inner speech (Fernyhough and Borghi, 2023) during the thinking period. The inner speech itself is often dialogic, i.e., it speaks to the self within the brain without any voice production (Alderson-Day et al., 2016). During the inner speech thinking period, the brain continues its attention to the inner world because of the processing of the internal voice.

Vocal language communication relies on the human ability to understand what the speaker is saying. Based on the auditory input pattern of vocal words, the human brain can recognize the meaning of words. If the vocal word is the name of an object, the human brain can recollect the multisensory imagery of the object simply by hearing the word. It is not clear yet how the cortical network recognizes the meaning of the word. It should be noted that recollection of multisensory imagery is possible only after the human brain has established associative learning of the vocal word with the multisensory cognitive imagery of an object.

We speculate that before the word-object associative learning, the vocal-word input activates a subset of pyramidal cells in the auditory cortex (anterior primary auditory cortex and anterior superior temporal gyrus) but cannot generate the multisensory imagery of an object in the higher cognitive areas (middle temporal gyrus and temporal pole gyrus) in the auditory ventral stream (Hickok and Poepel, 2007; Poliva, 2016). We speculate that during word-object associative learning, pyramidal-cell firings induced by feedforward auditory input interact with firings caused by subsequent top-down cognitive scene-signals of the object in the auditory cortex and higher cognitive areas (Figure 2). This interaction may cause a long-term enhancement in the synaptic connections between the vocal-word responsive pyramidal cells in the auditory cortex and pyramidal cells responsible for generating cognitive scene signals in higher cognitive areas. These proposed hypotheses are an extrapolation of our previous hypothesis of odor-object associative learning, i.e., odor-object associative learning occurs over the repeated respiratory cycles involving both inhalation (processing of feedforward odor information) and exhalation (incorporating top-down cognitive signals of objects) in the network connecting the olfactory cortex and higher cognitive areas (Mori and Sakano, 2025).

After establishing associative learning, feedforward transmission of auditory signals of vocal words can generate associated cognitive imagery of an object in the higher cognitive areas, resulting in cognition of word meaning. Furthermore, after establishing associative learning, recollection of cognitive imagery of the object can induce recall of auditory imagery of the word. Such internal recollection of words may help to produce vocal words for speaking.

## Discussion

Animals, including humans, live in both the outer and inner worlds. In the outer world, they search for food, detect danger, and find mating partners for their survival. Animals also pay attention to the inner world. They detect physiological changes in the body and evaluate the current status of themselves. They implement corrective action to restore the system to its intended state when the situation deviates in a negative direction. Thus, the animals have two cognitive worlds, both outside and inside of the body. In this article, we discussed how these two worlds are recognized, how the two sets of sensory information are transmitted to the cognitive areas, and how the current situation is evaluated for behavioral decisions. We also discussed how attention is directed to the objects in the outer and inner worlds. External attention is directed to the exteroceptive sensory information of the outer world, whereas internal attention is directed not only to the interoceptive sensory information, but also to the top-down cognitive/emotional scene-information generated internally by the cortical network (Chun et al., 2011).

Our sensory systems detect the changes in the surrounding world by the olfactory, taste, visual, auditory, and somatosensory systems. In addition, animals possess the navigation system that detects positional information of their location and movement (Moser et al., 2008; Hartley et al., 2013; Flossmann and Rochefort, 2021). In the outer world, it can tell their movement and distance from the object. In humans, this system tells us our present position and distance from the goal in the inner world. When the first-person self looks at the current status of the third-person self, we ask whether the situation is satisfactory or not by measuring the distance from the goal, i.e., the situation of what it should be. Our attention directed at the difference between the real and ideal worlds motivates us to take appropriate actions to improve the situation, satisfy ourselves, or recover our physical condition.

Language in humans facilitates smooth social interactions with other individuals and is quite useful in sharing beneficial information within a community to get food and avoid danger. Humans deliver speech by activating two separate motor systems, i.e., the emotional motor system and somatic motor system (Holstege and Subramanian, 2016). Mammals including humans generate exhalation-coupled vocalization by activating the emotional motor system containing the pathway-link of prefrontal cortex → PAG → nucleus retroambiguus (NRA) → motoneurons that are involved in vocalization. The emotional motor system includes autonomic and respiratory motor systems and is directly controlled by the higher cognitive areas for the inner world (Figure 2, a red box). In the same exhalation period, humans vocalize a variety of words in the sentence by activating the somatic motor system consisting of the motor and premotor cortices (Figure 2, MC), and brainstem motor neurons that innervate muscles for the mouth, oral, throat, and tongue movements. The higher cognitive areas for the outer world (Figure 2, a blue box) may exert direct control to the somatic motor system to articulate words and generate speech content. Thus, in humans, speech generation requires intimate interactions between the two cognitive worlds: higher cognitive areas for the outer world and that of the inner world (Figure 2, a pink double-headed arrow).

Language plays an important role not only in communicating with others in the outside world, but also in talking to ourselves in the cognitive world. This talking to the self by inner speech is helpful in constructing behavioral strategies to respond to the changes in the surrounding world. Animals, even without language, are able to predict what is likely to happen based on learned memory recollecting the associated scene in the previous experience. However, humans are able to formulate various possibilities by thinking. We as a first-person talk to the third-person self to “think” what to do, evaluating the situation, solving the problem, and aiming the goal to improve the current status. This self-conversation in the cognitive world appears to be unique to humans. Thinking facilitates our prediction of what is going to happen in the near future in the surrounding world, both inside and outside the body. This advanced ability of prediction makes us think “What are we, and where are we going?” as Paul Gauguin asked. Talking to ourselves makes self-cognition clear, recognizing ourselves as an object in both the outer and inner worlds. René Descartes said, “I think, therefore, I am.” By thinking, we realize the presence of ourselves in the cognitive world. Although other animals have awareness when they are awake, they probably do not possess clear cognition of the self without talking to themselves. Thus, self-cognition may be unique to humans who have a talking tool to “think.” Thus, thinking (inner speech) appears to be essential for us to develop science, art and other cultural activities. Language is also helpful in distributing useful information and culture to other individuals in the community.

Recent advances in artificial intelligence (AI) are quite remarkable. Although AI can process a large amount of information quickly, it is still a high-speed computer commanded by man-made programs. It has been discussed how AI can be humanized (Hassabis et al., 2017). We may be able to answer this question by asking what the difference between AI and humans is, or what is missing in AI compared with humans. Obvious differences are self-cognition and self-motivation in making decisions. AI at present does not cognize the self nor possess self-motivation. Then, how can one add these functions to AI? In this perspective article, we have discussed cognition of the self in both outer and inner worlds and considered possible neural pathways to make self-motivated output decisions. These studies will not only give new insights into our understanding of neural networks for decision making but also shed light on the humanization of AI.

## Data Availability

The original contributions presented in the study are included in the article/supplementary material, further inquiries can be directed to the corresponding authors.

## Glossary

ACA, anterior cingulate area

ACAd, dorsal part of the anterior cingulate area

ACAv, ventral part of the anterior cingulate area

AID, dorsal agranular insular cortex

AIV, ventral agranular insular cortex

AIP, posterior agranular insular cortex

AONdm, dorsomedial part of the anterior olfactory nucleus

AONl, lateral part of the anterior olfactory nucleus

AONm, medial part of the anterior olfactory nucleus

APC, anterior piriform cortex

AUD, auditory cortex

AUDp, primary auditory cortex

AUDs, secondary auditory cortex

Aud, auditory input

Amyg, amygdala

aIC, anterior insular cortex

BA, basal nucleus of amygdala

CA1, field CA1 of the hippocampus

CoA, cortical nucleus of amygdala

DP, dorsal peduncular cortex

ENT, entorhinal cortex

ENTl, lateral entorhinal cortex

ENTm, medial entorhinal cortex

Ect, ectorhinal cortex

FF, feedforward transmission of sensory signals

FrA, frontal association cortex

GU, gustatory cortex

Gus, gustatory input

Hippo, hippocampus

IL, infralimbic cortex

Lat Olf, lateral olfactory input

Lat Pain, lateral pain input

MC, motor cortex

Med Olf, medial olfactory input

Med Pain, medial pain input

MPtA, medial parietal association cortex

Mrgprd, MAS-related G-protein coupled receptor member D

MTh, medial thalamic nuclei

M1, primary motor cortex

M2, secondary motor cortex

OBl, lateral map of the olfactory bulb

OBm, medial map of the olfactory bulb

OE, olfactory epithelium

Oral SS, oral tactile input

ORBdl, dorsolateral orbitofrontal cortex

ORBl, lateral orbitofrontal cortex

ORBm, medial orbitofrontal cortex

ORBvl, ventrolateral orbitofrontal cortex

PAG, periaqueductal gray

PBel, external lateral nucleus of parabrachial nuclei

PBsl, superior lateral nucleus of the parabrachial nuclei

PRh, perirhinal cortex

PrL, prelimbic cortex

PVL, ventral posterolateral thalamic nucleus

PVT, paraventricular thalamic nucleus

RSA, retrosplenial agranular cortex

RSG, retrosplenial granular cortex

SC, spinal cord

SN, salience network

SS, somatosensory input

SSp, primary somatosensory cortex

SSrt*, rostro-lateral part of the somatosensory cortex

SSs, secondary somatosensory cortex

SUB, subiculum

TRPV1, Transient receptor potential cation channel subfamily V member 1

TeA, temporal association cortex

TT, tenia tecta

TTd, dorsal tenia tecta

TTv, ventral tenia tecta

VIS, visual cortex

VISp, primary visual cortex

VISs, secondary visual cortex

Vis, visual input

VISC, visceral cortex

Visc, visceral input

## References

[B1] Aguilar D. D. McNally J. M. (2022). Subcortical control of the default mode network: role of the basal forebrain and implications for neuropsychiatric disorders. Brain Res. Bull. 185, 129–139. 10.1016/j.brainresbull.2022.05.005 35562013 PMC9290753

[B2] Alderson-Day B. Weis S. McCarthy-Jones S. Moseley P. Smailes D. Fernyhough C. (2016). The brain's conversation with itself: neural substrates of dialogic inner speech. Social Cogn. Affect. Neurosci., 2016, 110–120. 10.1093/scan/nsv094 26197805 PMC4692319

[B3] Azzalini D. Rebollo I. Tallon-Baudry C. (2019). Visceral signals shape brain dynamics and cognition. Trends Cogn. Sci. 23, 488–509. 10.1016/j.tics.2019.03.007 31047813

[B4] Behan M. Haberly L. B. (1999). Intrinsic and efferent connections of the endopiriform nucleus in rat. J. Comp. Neurol. 408, 532–548. 10.1002/(SICI)1096-9861(19990614)408:4<532::AID-CNE7>3.0.CO;2-S 10340503

[B5] Behbeharni M. N. (1995). Functional characteristics of the midbrain periaqueductal gray. Prog. Neurobiol. 46, 575–605. 10.1016/0301-0082(95)00009-K 8545545

[B6] Buckner R. L. Andrews-Hanna J. R. Schacter D. L. (2008). The brain's default network: anatomy, function, and relevance to disease. Ann. N.Y. Acad. Sci. 1124, 1–38. 10.1196/annals.1440.011 18400922

[B7] Bushnell M. C. Ceko M. Low L. A. (2013). Cognitive and emotional control of pain and its disruption in chronic pain. Nat. Rev. Neurosci. 14, 502–511. 10.1038/nrn3516 23719569 PMC4465351

[B8] Chao O. Y. de Souza Silva M. A. Yang Y.-M. Huston J. (2020). The medial prefrontal cortex – hippocampus circuit that integrates information of object, place and time to construct episodic memory in rodents: behavioral, anatomical and neurochemical properties. Neurosci. Biobehv. Rev. 113, 373–407. 10.1016/j.neubiorev.2020.04.007 32298711 PMC7302494

[B9] Chun M. M. Golomb J. D. Turk-Browne N. B. (2011). A taxonomy of external and internal attention. Annu. Rev. Psychol. 62, 73–101. 10.1146/annurev.psych.093008.100427 19575619

[B10] Craig A. D. (2002). How do you feel? Interoception: the sense of the physiological condition of the body. Nat. Rev. Neurosci. 3, 655–666. 10.1038/nrn894 12154366

[B11] De Ridder D. Vanneste S. Smith M. Adhia D. (2022). Pain and the triple network model. Front. Neurol. 13:75724. 10.3389/fneur.2022.757241 35321511 PMC8934778

[B12] Demartsev V. Manser M. B. Tattersall G. (2022). Vocalization-associated respiratory patterns: thermography-based monitoring and detection of preparation for calling. J. Exp. Biol. 225:jeb243474. 10.1242/jeb.243474 35142353 PMC8976942

[B13] Do J. P. Xu M. Lee S.-H. Chang W.-C. Zhang S. Chung S.. (2016). Cell type-specific long-range connections of basal forebrain circuit. Elife 5:e13214. 10.7554/eLife.22475 27642784 PMC5095704

[B14] Fernyhough C. Borghi A. M. (2023). Inner speech as language process and cognitive tool. Trends Cogn. Sci. 27, 1180–1193. 10.1016/j.tics.2023.08.014 37770286

[B15] Flossmann T. Rochefort N. L. (2021). Spatial navigating signals in rodent visual cortex. Curr. Opin. Neurobiol. 67, 163–173. 10.1016/j.conb.2020.11.004 33360769

[B16] Fox M. D. Snyder A. Z. Vincent J. L. Corbetta M. Van Essen D. C. Raichle M. E. (2005). The human brain is intrinsically organized into dynamic, anticorrelated functional networks. Proc. Natl. Acad. Sci. U.S.A. 102, 9673–9678. 10.1073/pnas.0504136102 15976020 PMC1157105

[B17] Gallagher S. (2000). Philosophical conceptions of the self: implications for cognitive science. Trends Cogn. Sci. 4, 14–21. 10.1016/S1364-6613(99)01417-5 10637618

[B18] Gangopadhyay P. Chawla M. Dal Monte O. Chang S. W. C. (2021). Prefrontal – amygdala circuits in social decision making. Nat. Neurosci. 24, 5–18. 10.1038/s41593-020-00738-9 33169032 PMC7899743

[B19] Goulden N. Khusnulina A. Davis N. J. Bracewell R. M. Bokde A. L. McNulty J. P.. (2014). The salience network is responsible for switching between the default mode network and the central executive network: replication from DCM. Neuroimage 99, 180–190. 10.1016/j.neuroimage.2014.05.052 24862074

[B20] Greicius M. D. Krasnow B. Reiss A. L. Menon V. (2003). Functional connectivity in the resting brain: a network analysis of the default mode hypothesis. Proc. Natl. Acad. Sci. U.S.A. 100, 253–258. 10.1073/pnas.0135058100 12506194 PMC140943

[B21] Griffin A. (2015). Role of the thalamic nucleus reuniens in mediating interactions between the hippocampus and medial prefrontal cortex during spatial working memory. Front. Syst. Neurosci. 9:29. 10.3389/fnsys.2015.00029 25805977 PMC4354269

[B22] Hartley T. Lever C. Burgess N. O'Keefe J. (2013). Space in the brain: how the hippocampal formation supports spatial cognition. Philos. Trans. R. Soc. B. Biol. Sci. 369:20120510. 10.1098/rstb.2012.0510 24366125 PMC3866435

[B23] Hassabis D. Kumaran D. Summerfield C. Botvinick M. (2017). Neuroscience-inspired artificial intelligence. Neuron 95, 245–258. 10.1016/j.neuron.2017.06.011 28728020

[B24] Hickok G. Poepel D. (2007). The cortical organization of speech processing. Nat. Rev. Neurosci. 8, 393–402. 10.1038/nrn2113 17431404

[B25] Holstege G. (2014). The periaqueductal gray controls brainstem emotional motor systems including respiration. Prog. Brain Res. 209, 379–405. 10.1016/B978-0-444-63274-6.00020-5 24746059

[B26] Holstege G. Subramanian H. H. (2016). Two different motor systems are needed to generate human speech. J. Comp. Neurol. 524, 1558–1577. 10.1002/cne.23898 26355872

[B27] Huang T. Lin S-H Malewicz M. N. Zhang Y. Zhang Y. Goulding M. LaMotte R. H.. (2019). Identification of pathways required for sustained pain-associated coping behaviors Nature 565, 86–90. 10.1038/s41586-018-0793-8 30532001 PMC6461409

[B28] Jürgens U. (2002). Neural pathways underlying vocal control. Neurosci. Biobehav. Rev. 26, 235–258. 10.1016/S0149-7634(01)00068-9 11856561

[B29] Katsuki F. Constantinidis C. (2014). Bottom-up and top-down attention: different processes and overlapping neural systems. Neuroscientist 20, 509–521. 10.1177/1073858413514136 24362813

[B30] Kobayakawa K. Kobayakawa R. Matsumoto H. Oka Y. Imai T. Ikawa M.. (2007). Innate versus learned odor processing in the mouse olfactory bulb. Nature 450, 503–508. 10.1038/nature06281 17989651

[B31] Kulkarni B. Bentley D. E. Elliot. R Youell P. Watson A. Derbyshire S. W. G.. (2005). Attention to the pain localization and unpleasantness discriminates the functions of the medial and lateral pain systems. Eur. J. Neurosci. 21, 3133–3142. 10.1111/j.1460-9568.2005.04098.x 15978022

[B32] Marek R. Strobel C. Bredy T. W. Sah P. (2013). The amygdala and medial prefrontal cortex: partners in the fear circuit. J. Physiol. 591, 2381–2391. 10.1113/jphysiol.2012.248575 23420655 PMC3678031

[B33] McKlveen J. M. Myers B. Herman J. P. (2015). The medial prefrontal cortex: coordinator of autonomic, neuroendocrine and behavioral responses to stress. J. Neuroendocrinol. 27, 446–456. 10.1111/jne.12272 25737097 PMC4580281

[B34] Menon V. (2023). 20 years of the default mode network: a review and synthesis. Neuron 111, 2469–2487. 10.1016/j.neuron.2023.04.023 37167968 PMC10524518

[B35] Menon V. Cerri D. Lee B. Yuan R. Lee S.-H. Shih Y.-Y. I. (2023). Optogenetic stimulation of anterior insular cortex neurons in male rats reveals causal mechanisms underlying suppression of default mode network by salience network. Nat. Commun. 14:866. 10.1038/s41467-023-36616-8 36797303 PMC9935890

[B36] Menon V. Uddin L. Q. (2010). Saliency, switching, attention and control: a network model of insula function. Brain Struct. Funct. 214, 655–667. 10.1007/s00429-010-0262-0 20512370 PMC2899886

[B37] Mori K. Sakano H. (2011). How is the olfactory map formed and interpreted in the mammalian brain? Annu. Rev. Neurosci. 34, 467–499. 10.1146/annurev-neuro-112210-112917 21469960

[B38] Mori K. Sakano H. (2021). Olfactory circuitry and behavioral decisions. Annu. Rev. Physiol. 83, 231–256. 10.1146/annurev-physiol-031820-092824 33228453

[B39] Mori K. Sakano H. (2022a). Processing of odor information during the respiratory cycle in mice. Font. Neural Circuits 16:861800. 10.3389/fncir.2022.861800 35431818 PMC9008203

[B40] Mori K. Sakano H. (2022b). Neural circuitry for stress information of environmental and internal odor. Front. Behav. Neurosci. 16:943647. 10.3389/fnbeh.2022.943647 35783233 PMC9245520

[B41] Mori K. Sakano H. (2024a). Circuit formation and sensory perception in the mouse olfactory system. Front. Neural Circuits 18:1342576. 10.3389/fncir.2024.1342576 38434487 PMC10904487

[B42] Mori K. Sakano H. (2024b). One respiratory cycle as a minimum time unit for making behavioral decisions in the mammalian olfactory system. Front. Neurosci. 18:1423694. 10.3389/fnins.2024.1423694 39315076 PMC11417025

[B43] Mori K. Sakano H. (2025). Associative learning and recollection of olfactory memory during the respiratory cycle in mammals: how is the self cognized in consciousness? Front. Neurosci. 18:1513396. 10.3389/fnins.2024.1513396 39897952 PMC11783145

[B44] Moser E. I. Kropff E. Moser M. B. (2008). Place cells, grid cells, and the brain's spatial representation system. Annu. Rev. Neurosci. 31, 69–89. 10.1146/annurev.neuro.31.061307.090723 18284371

[B45] Nani A. Manuello J. Mancuso L. Liloa D. Costa T. Cauda F. (2019). The neural correlates of consciousness and attention: two sister processes of the brain. Front. Neurosci. 13:1169. 10.3389/fnins.2019.01169 31749675 PMC6842945

[B46] Narikiyo K. Manabe H. Yoshihara Y. Mori K. (2018). Respiration-phased switching between sensory inputs and top-down inputs in the olfactory cortex. bioRxiv. 10.1101/499749

[B47] Narikiyo K. Mizuguchi R. Ajima A. Shiozaki M. Hamanaka H. Johansen J. P.. (2020). The claustrum coordinates cortical slow-wave activity. Nat. Neurosci. 23, 741–753. 10.1038/s41593-020-0625-7 32393895

[B48] Poliva O. (2015). From where to what: a neuroanatomically based evolutionary model of the emergence of speech in humans. F10000Research 4:67. 10.12688/f1000research.6175.1 PMC5600004 28928931

[B49] Poliva O. (2016). From mimicry to language: a neuroanatomically based evolutionary model of the emergence of vocal language. Front. Neurosci. 10:307. 10.3389/fnins.2016.00307 27445676 PMC4928493

[B50] Price J. L. (1999). Prefrontal cortical networks related to visceral function and mood Ann. N.Y. Acad. Sci. 877, 383–396. 10.1111/j.1749-6632.1999.tb09278.x 10415660

[B51] Raichle M. E. MacLeod A. M. Snyder A. Z. Powers W. J. Gusnard D. A. Shulman G. L. (2001). A default mode of brain function. Proc. Natl. Acad. Sci. U.S.A. 98, 676–682. 10.1073/pnas.98.2.676 11209064 PMC14647

[B52] Saito H. Nishizumi H. Suzuki S. Matsumoto H. Ieki N. Abe T.. (2017). Immobility responses are induced by photoactivation of single glomerular species responsive to fox odour TMT. Nat. Commun. 8:16011. 10.1038/ncomms16011 28685774 PMC5504302

[B53] Seeley W. W. Menon V. Schatzberg A. F. Keller J. Glover G. H. Kenna H.. (2007). Dissociable intrinsic connectivity networks for salience processing and executive control. J. Neurosci. 27, 2349–2356. 10.1523/JNEUROSCI.5587-06.2007 17329432 PMC2680293

[B54] Seyfarth R. M. Cheney D. L. (2010). Production, usage, and comprehension in animal vocalizations. Brain Lang. 115, 92–100. 10.1016/j.bandl.2009.10.003 19944456

[B55] Skog T. D. Jhonson S. B. Hinz D. C. Lingg R. T. Schulz E. N. Luna J. T.. (2024). A prefrontal → periaqueductal gray pathway differentially engages autonomic, hormonal, and behavioral features of the stress-coping response. J. Neurosci. 44:e0844242024. 10.1523/JNEUROSCI.0844-24.2024 39313320 PMC11561873

[B56] Sridharan D. Levitin D. J. Menon V. (2008). A critical role for the right fronto-insular cortex in switching between central-executive and default-mode networks. Proc. Natl. Acad. Sci. U.S.A. 105, 12569–12574. 10.1073/pnas.0800005105 18723676 PMC2527952

[B57] Takahashi D. Y. Narayanan D. Z. Ghazanfar A. A. (2013). Coupled oscillator dynamics of vocal turn-taking in monkeys. Curr. Biol. 23, 2162–2168. 10.1016/j.cub.2013.09.005 24139740

[B58] Tovote P. Esposito M. S. Botta P. Chaudun F. Fadok J. P. Markovic M.. (2016). Midbrain circuits for defensive behaviour. Nature 534, 206–212. 10.1038/nature17996 27279213

[B59] Villano I. Messina A. Valenzano A. Moscatelli F. Esposito T. Monda V.. (2017). Basal forebrain cholinergic system and orexin neurons: effects on attention. Front. Behav. Neurosci. 11:10. 10.3389/fnbeh.2017.00010 28197081 PMC5281635

[B60] Vincent J. L. Kahn I. Snyder A. Z. Raichle M. E. Buckner R. L. (2008). Evidence for a frontoparietal control system revealed by intrinsic functional connectivity. J. Neurophysiol. 100, 3328–3342. 10.1152/jn.90355.2008 18799601 PMC2604839

[B61] Wang K. L. L. Nair A. Augustine G. J. (2021). Changing the cortical conductor's tempo: neuromodulation of the claustrum. Front. Neural Circuits 15:658228. 10.3389/fncir.2021.658228 34054437 PMC8155375

[B62] Wang L.-B. Su X.-J. Wu Q.-F. Xu X. Wang X.-Y. Chen M.. (2022). Pathways for transmitting reflexive and affective dimensions of nocifensive behaviors evoked by selective activation of Mas-related G protein-coupled receptor D-positive and transient receptor potential vanilloid 1-positive subsets of nociceptors. Front. Cell. Neurosci. 16:910670. 10.3389/fncel.2022.910670 35693883 PMC9175034

[B63] Wercberger R. Basbaum A. I. (2019). Spinal cord projection neurons: a superficial, and also deep analysis. Curr. Opin. Physiol. 11, 109–115. 10.1016/j.cophys.2019.10.002 32864531 PMC7450810

[B64] Xu M. Chung S. Zhang S. Zhong P. Ma C. Chang W.-C.. (2015). Basal forebrain circuit for sleep-wake control. Nat. Neurosci. 18, 1641–1647. 10.1038/nn.4143 26457552 PMC5776144

[B65] Zaidel A. Salmon R. (2023). Multisensory decisions from self to world. Trans. R. Soc. B. 378:20220335. 10.1098/rstb.2022.0335 37545311 PMC10404927

[B66] Zhang Y. S. Ghazanfar A. A. (2020). A hierarchy of autonomous systems for vocal production. Trends Neurosci. 43, 115–126. 10.1016/j.tins.2019.12.006 31955902 PMC7213988

[B67] Zingg B. Hintiryan H. Gou L. Song M. Y. Bay M. Bienkowski M.. (2012). Neural network of the mouse neocortex. Cell 156, 1096–1111. 10.1016/j.cell.2014.02.023 24581503 PMC4169118

